# Canadian survey on pandemic flu preparations

**DOI:** 10.1186/1471-2458-10-125

**Published:** 2010-03-11

**Authors:** Paul Ritvo, Kumanan Wilson, JL Gibson, C Guglietti, CS Tracy, JX Nie, AR Jadad, REG Upshur, the University of Toronto Joint Centre for Bioethics Pandemic Ethics Working Group

**Affiliations:** 1 School of Kinesiology and Health Science, York University, 4700 Keele Street, Toronto, M3J 1P3, Canada; 2 Ottawa Health Research Institute, Department of Medicine, University of Ottawa, 725 Parkdale Ave, Ottawa, K1Y 4E9, Canada; 3 University of Toronto Joint Centre for Bioethics, 155 College, Street, Suite 754, Toronto, M5T 1P8, Canada; 4 Primary Care Research Unit, Sunnybrook and Women's College Health, Sciences Centre, 2075 Bayview Avenue, Toronto, M4N 3M5, Canada; 5 Centre for Global eHealth Innovation, Toronto General Hospital, R Fraser Elliott Building, 4th Floor, 190 Elizabeth Street, Toronto, M5G 2C4, Canada; 6 Department of Family and Community Medicine, University of Toronto, 263 McCaul Street, 3rd Floor, Toronto, M5T 1W7, Canada; 7 Department of Health Policy, Management, & Evaluation, University of Toronto, 155 College Street, 4th Floor, Toronto, M5T 1P8

## Abstract

**Background:**

The management of pandemic influenza creates public health challenges.

An ethical framework, 'Stand on Guard for Thee: ethical considerations in pandemic influenza preparedness' that served as a template for the World Health Organization's global consultation on pandemic planning, was transformed into a survey administered to a random sample of 500 Canadians to obtain opinions on key ethical issues in pandemic preparedness planning.

**Methods:**

All framework authors and additional investigators created items that were pilot-tested with volunteers of both sexes and all socioeconomic strata. Surveys were telephone administered with random sampling achieved via random digit dialing (RDD). Eligible participants were adults, 18 years or older, with per province stratification equaling provincial percent of national population. Descriptive results were tabulated and logistic regression analyses were used to assess whether demographic factors were significantly associated with outcomes.

**Results:**

5464 calls identified 559 eligible participants of whom 88.5% completed surveys. Over 90% of subjects agreed the most important goal of pandemic influenza preparations was saving lives, with 41% endorsing saving lives solely in Canada and 50% endorsing saving lives globally as the highest priority. Older age (OR = 8.51, p < 0.05) and current employment (OR = 9.48, p < 0.05) were associated with an endorsement of saving lives globally as highest priority. About 90% of respondents supported the obligation of health care workers to report to work and face influenza pandemic risks excepting those with a serious health condition that increased risks. Over 84% supported the government's provision of disability insurance and death benefits for health care workers facing elevated risk. Strong majorities favored stocking adequate protective antiviral dosages for all Canadians (92%) and, if effective, influenza vaccinations (95%). Over 70% agreed Canada should provide international assistance to poorer countries for pandemic preparation, even if resources for Canadians were reduced. While 92% of this group, believed provision should be 7 to 10% of all resources generated, 43% believed the provision should be greater than 10%.

**Conclusions:**

Results suggest trust in public health officials to make difficult decisions, providing emphasis on reciprocity and respect for individual rights.

## Background

Because influenza pandemics can cause significant morbidity, mortality and societal disruption and result in extraordinary demands on citizens and health care workers, deep ethical challenges are confronted in formulating pandemic responses [1]. This was illustrated during SARS in Canada in 2003. The use of restrictive measures for disease control, the reallocation of health care resources to an unknown infectious disease and obligations to ensure health care worker safety were complex, pressing ethical issues. SARS further reinforced the need to reflect on global obligations with regards to a possible SARS response [2].

The ethical concerns encountered in pandemic preparedness have been articulated in multiple documents. In 2005, the University of Toronto's Joint Centre for Bioethics published a report entitled Stand on Guard for Thee [2] which was influential in the WHO's global consultation on ethical issues, and in assisting pandemic response planners [3]. This report addressed ethical, legal, and social issues related to influenza response that revolved around four questions: 1) in a pandemic crisis, what obligations do health workers have to serve and how are healthcare systems obligated in relation to health workers providing service? 2) how should limited resources be allocated? 3) how should information be communicated to the public and who should lead public dialogue? 4) what global governance measures should be used to advance risk reducing activities internationally?

Public engagement on such issues and questions is essential in deriving adequate policy. The Eleventh Futures Forum (EFF) on the ethical governance of pandemic preparedness reinforced the need for public engagement and recommended a wide variety of engagement techniques [4]. As surveys are one means of determining and stimulating public opinion on ethically related issues, we transformed Stand on Guard for Thee into a survey instrument to better understand the opinions of Canadians.

In this paper, we report on survey outcomes and their implications for future frameworks. The results are of immediate importance given the global emergence of the H1N1 strain. We are unaware of any other representative survey of Canadian opinion spanning the range of ethical challenges in pandemic response.

## Methods

### Ethics

This study has been reviewed and approved by the University of Toronto and York University Research Ethics Boards.

### Survey Content

All original framework authors and additional investigators translated text from "Stand on Guard for Thee" into survey format. Decisions about item construction were based on investigator consensus. Once preliminary items were judged adequately conveyed via telephone interview, the survey was pilot-tested with volunteers of both sexes and all socioeconomic strata. Each pilot subject commented on item clarity and this feedback was reviewed by each investigator. Final items resulted from iterative revisions where face validity (in relation to the framework) was matched with 8th grade reading level to precisely convey content to a population varying in educational and ethnic background.

### Sampling

Random sampling was achieved via random digit dialing with random digits from across Canada purchased from Sampling Modeling and Research Technologies, Inc. (SMRT, Markham, Ontario). Eligible participants included adults, 18 years or older. Screening of participants was based on standardized inquiries regarding age, citizenship, provincial residency and gender. To characterize nationwide attitudes, we stratified the sample per province to obtain sub-samples equivalent to the province's contribution to national population. To maximize response rate, we administered a core of selected items to all respondents while administering additional sections (Form A and Form B) to alternate subjects. Each participant was provided the option of survey administration in English or French and all contacts with Quebec residents were initiated in French. The survey administration was conducted by a team of N = 7 graduate students (2 males, 5 females).

### Analysis

Descriptive results of the percentages of responses to the survey questions were tabulated. Logistic regression analyses were used to ascertain which demographic factors were significantly associated with survey outcomes. Specific factors were treated as independent (predictor) variables and key findings as dependent (outcome) variables. These analyses addressed the following factors: sex; age; educational background; employment status; residence (eastern provinces versus central provinces versus western provinces; urban versus rural area); relationship status (married or single) and family status (with children versus without children). Dichotomous variables (sex, residential community, and parity) were used in combination with continuous variables that were dichotomized as follows: age [18-50 years versus 51-93 years], martial status [married versus non-married (single, separated, divorced, widowed)], education (<college/university versus = college/university), and employment [employed (full time) versus unemployed (part time & unemployed)].

## Results

### Sampling

Of the 5464 calls made, 1690 calls reached answered telephones. Of the 559 individuals who answered calls and could be identified as eligible for participation, 88.5% completed the survey. The demographics of the final sample are presented in Table 1. Altogether, 501 subjects responded to common items, and 261 subjects responded to Form A items while 240 subjects responded to Form B items (Table 1). Demographics for the entire sample vary insignificantly from the demographics of the subsamples completing Form A and Form B.

**Table 1:** Demographical information of participants

Demographic Variable	Mean (SD)	Total Sample Frequency (%)	Form A Frequency	Form B Frequency	Refused
**Age - Years**	51(17)		52(17)	49(16)	

**Sex**		**501**	**259**	**242**	
Male		176(35%)	90(34%)	86(35%)	
Female		325(65%)	169(66%)	156(65%)	

**Marital Status**		**493**	**256**	**237**	**8**
Single		107(22%)	54(22%)	53(22%)	
Married		311(63%)	160(63%)	151(63%)	
Widowed		30(6%)	19(7%)	11(6%)	
Separated		14(3%)	7(2%)	7(3%)	
Divorced		31(6%)	15(6%)	15(6%)	

**Parity**		**494**	**257**	**237**	**7**
Children		376(76%)	198(76%)	178(76%)	
No Children		118(24%)	59(24%)	59(24%)	

**Education**		**492**	**254**	**238**	**9**
< High School		39(8%)	20(8%)	19(8%)	
High School		126(26%)	72(28%)	54(23%)	
Some College		49(10%)	26(10%)	23(10%)	
College		97(20%)	42(17%)	55(23%)	
University		135(27%)	70(28%)	65(27%)	
Masters		36(7%)	19(7%)	17(7%)	
Phd		10(2%)	5(2%)	5(2%)	

**Employment**		**495**	**257**	**238**	**6**
Full time		261(53%)	141(55%)	120(53%)	
Part time		53(11%)	23(9%)	30(11%)	
Not employed		181(37%)	93(36%)	88(37%)	

**Ethnicity**		**496**	**257**	**239**	**5**
Caucasian		407(82%)	205(80%)	202(85%)	
Asian		21(4%)	12(5%)	9(4%)	
Hispanic		8(2%)	5(2%)	3(1%)	
Canadian African		19(4%)	12(5%)	7(3%)	
Canadian		2(>1%)	2(1%)	0(0%)	
Caribbean		11(2%)	7(3%)	4(6%)	
Aboriginal Other		28(6%)	14(5%)	14(6%)	

**Residential**		**497**	**259**	**238**	**4**
Urban		303(61%)	158(61%)	145(61%)	
Rural		194(39%)	101(39%)	93(39%)	

**Province**		**501**	**261**	**240**	
British Columbia		65(13%)	33(13%)	32(13%)	
Alberta		52(10%)	26(10%)	26(11%)	
Saskatchewan		14(3%)	5(2%)	9(4%)	
Manitoba		19(4%)	10(4%)	9(4%)	
Ontario		197(40%)	104(40%)	93(39%)	
Quebec		117(23%)	63(24%)	54(23%)	
Newfoundland		8(1%)	5(2%)	3(1%)	
New Brunswick		13(3%)	5(2%)	8(3%)	
Nova Scotia		14(3%)	8(3%)	6(2%)	
Prince Edward Island		2(<1%)	2(<1%)	0(0%)	

### Survey Findings

Full survey results are found in Table 2 and below is a summary reflecting findings with the highest percentages of support.

**Table 2:** Reported response frequencies per item addressing ethical issues

	Agree	Neutral	Disagree	Total	Refused
2a) Saving as many lives as possible, in Canada	483(97%)	7(1%)	10(2%)	500	1

2b) Saving as many lives as possible, globally	474(95%)	12(2%)	13(3%)	499	2

2c) Maintaining social order	447(90%)	34(7%)	16(3%)	497	4

2d) Protecting human rights	413(81%)	41(8%)	53(10%)	498	3

2e) Preventing economic decline	392(79%)	63(13%)	43(9%)	498	3

					

	Saving as many lives as possible, in Canada	Saving as many lives as possible, globally	Maintain social order	Protect human rights	Prevent economic decline

					

3) If one purpose for the Canadian Pandemic flu plan what would it be?	205(41%)	249(50%)	19(4%)	16(3%)	7(1.3%)

	**Agree**	**Neutral**	**Disagree**	**Total**	**Refused**

4) Health care workers should report to work and face all risks when caring for patients during a flu pandemic, providing precautions are taken to protect their safety	234(90%)	14(5%)	12(5%)	260	1

5) Health care workers who do not report to work during a pandemic should face loss of employment or loss of professional license	123(48%)	38(15%)	97(38%)	258	3

6) Health care workers who must care for young children or elderly relatives should not be expected to work during a pandemic	146(57%)	33(13%)	76(30%)	255	6

7) Governments should reserve the right to conscript health care workers during a pandemic	123(47%)	25(10%)	112(43%)	260	1

8) If a health care worker has a serious health condition that can increase their risk, they should not have to come to work during a pandemic	233(89%)	6(2%)	22(9%)	261	

9) Governments should provide disability insurance and death benefits at no charge for health care workers at risk during a pandemic flu crisis	221(85%)	17(7%)	23(9%)	261	

10) If a health care worker does not feel safe at work, he or she should be able to file a grievance without fear of consequences	218(84%)	21(8%)	22(8%)	261	

11) It is reasonable for government to have the power to order quarantine during a pandemic flu outbreak and to suspend other rights, like the right to assemble or travel without restriction	222(85%)	23(9%)	15(6%)	260	1

12) People who do not agree with their quarantine order should be able to ask government officials to review the quarantine order and end it	162(63%)	21(8%)	76(29%)	259	2

14) The government should ensure that people in quarantine have their basic needs met, like food, shelter, and social support	246(95%)	7(2.5%)	7(2.5%)	260	1

15) After the quarantine is over, the government should provide support services, like counseling, for people who were in quarantine	206(79%)	27(10%)	28(11%)	261	

16) If successful, the pandemic flu vaccine should be made freely available to every Canadian resident, including adults and children.	227(95%)	8(3%)	5(2%)	240	

17) There should be adequate amounts of antiviral medications provided to every Canadian	221(92%)	9(4%)	10(4%)	240	

22) Wealthy countries like Canada should provide international assistance to help poorer countries prepare for a pandemic, even if that reduces the resources available to Canadians	167(70%)	28(12%)	45(18%)	240	

24) Countries should have the right to close their orders to travelers coming from areas where outbreaks have occurred, even when the travelers are the own citizens.	179(75%)	22(10%)	39(15%)	240	

25) International authorities should advise against travel to outbreak areas to stop a pandemic from spreading, even when this results in serious economic losses	234(99%)	6(1%)	0(0%)	240	

27) Because during a pandemic, key personnel, like first responders, may be overwhelmed by the catastrophe, a plan should be developed to enable members of the public to play a role in maintaining order and offering services in some cases with proper training.	489(98%)	5(1%)	5(1%)	499	2

28a) How would you like risks to be communicated to you - By radio?	451(90%)	18(4%)	32(6%)	501	

28b) During a pandemic, it will be likely to communicate important health risks to the public. How would you like risks to be communicated to you - By TV?	486(97%)	11(2%)	4(1%)	501	

28c). How would you like risks to be communicated to you - By internet?	408(81%)	43(9%)	50(10%)	501	

28d) How would you like risks to be communicated to you - By telephone?	347(69%)	65(13%)	89(18%)	501	

29) What risks would you like information about - Where the epidemic is most active?	487(98%)	7(1%)	5(1%)	499	2

30). What risks would you like information about - Risk of death	347(69%)	70(14%)	83(17%)	500	1

31) What risks would you like information about - Risk of infection	390(78%)	50(10%)	60(12%)	500	1

					

	Parking in a no parking zone	Speeding on a busy street	Physical assault	Man-slaughter	

32) Disobeying a quarantine order is most like which of the four following alternatives?	16 (6%)	47 (17%)	72 (27%)	131 (50%)	

### Purpose of Pandemic Influenza Preparation

**Figure 1:**
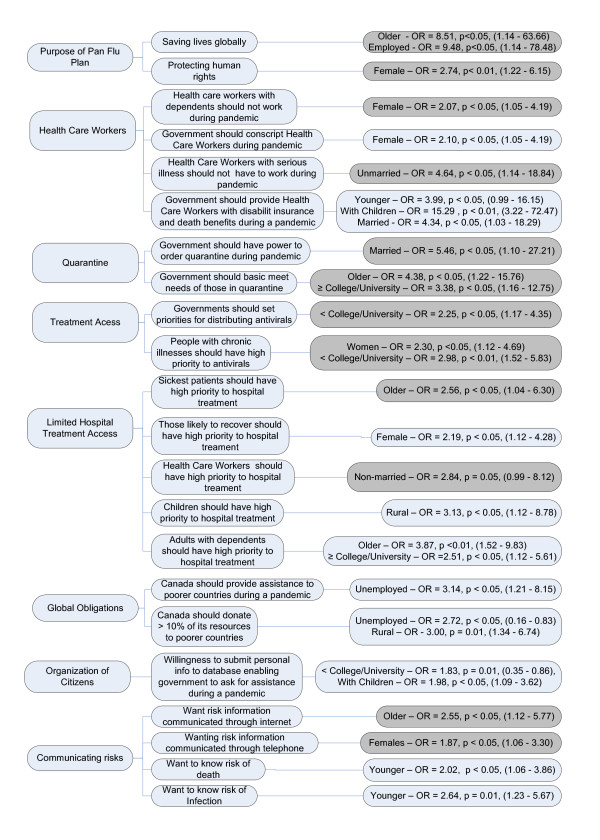
**Demographic variables significantly associated with agreement (dark grey) and disagreement (light grey) with ethical items and associated Odds Ratios, significance levels and confidence intervals**.

About 91% of subjects agreed the most important goal of pandemic influenza preparations was saving lives, with 41% endorsing saving lives solely in Canada as the highest priority and 50% endorsing the saving of lives globally as the highest priority. Older age (aged 51 - 93 years (OR = 8.51, p < 0.05)) and current employment (as opposed to unemployment) (OR = 9.48, p < 0.05) were associated with an endorsement of saving lives globally as highest priority (Figure 1) while female gender was associated (OR = 2.74, p = 0.01) with an endorsement of the protection of human rights as an important priority. Strong majorities also favored stocking adequate protective antiviral dosages for all Canadians (92%) and, if effective, influenza vaccinations (95%).

### Healthcare Workers Obligations

About 90% of respondents supported the obligation of health care workers to report to work and face associated risks during an influenza pandemic unless they had a serious health condition that increased their risks. Over 84% supported the government's provision of disability insurance & death benefits at no charge for health care workers facing elevated risk. Other strong findings indicated support for health care workers who don't feel safe at work being able to file a grievance (84%) and for a plan enabling members of the public to play a volunteer role in maintaining order and offering services should first responders be overwhelmed (97%).

Majorities were found for the right of governments to conscript health care workers during a pandemic (62% agree versus 25% disagree versus 13% neutral) and for the rights of health care workers caring for children or elderly relatives to be excused from pandemic duties (57% agree versus 30% disagree versus 33% neutral). Despite these concessions, a modest majority favored loss of employment or professional license (48% agree versus 37% disagree versus 15% neutral), if health workers were absent without cause during a pandemic.

In terms of regression findings, female gender was associated with agreeing that health care workers with dependents should not have to report to work (OR = 2.07, p < 0.05) (Figure 1) and with not agreeing (OR = 2.10, p < 0.05) the government should be able to conscript health care workers during a pandemic (Figure 1). Unmarried individuals (OR = 4.64, p < 0.05) were more likely to agree that health care workers with serious health conditions should not have to work during a pandemic and younger respondents of both genders (OR = 3.99, p = 0.05), those with children (OR = 15.29, p < 0.001), and those married (OR = 4.34, p < 0.05) were more likely to disagree that the government should provide disability insurance and death benefits to health care workers during a pandemic.

### Quarantine and Other Restrictive Measures

Strong majorities favored governmental power to order quarantines and suspend rights (e.g. traveling, right to assemble) during outbreaks (85%), providing those quarantined had basic needs met (food, shelter, social support) (95%) and access to support services after the end of quarantine (79%). This advocacy of governmental power extended to international authorities advising against travel to outbreak areas to stop a pandemic spread, even when the consequences included serious economic loss (97%). Non-married participants were more likely to endorse (OR = 5.46, p < 0.05) the government's power to order quarantines while older subjects (OR = 4.38, p < 0.05) and those with = college education (OR = 3.38, p < 0.03) were more likely to endorse the government's responsibility to meet the basic needs of those quarantined.

### Resource Allocation

Strong findings were observed for how priorities should be determined for antiviral treatments, if adequate stores were unavailable for all Canadians; and how priorities should be apportioned for hospital treatment, if adequate facilities were unavailable for all (Table 3). In both situations, the highest priorities were assigned to children and health workers infected while serving patients.

**Table 3:** Response frequencies for priority setting items

*Access to Antiviral Medication*	High	%	Moderate	%	Low	%	Total	Refused
Children	215	90	22	9	3	1	240	

Seniors	115	48	84	35	41	17	240	

Health care workers	228	95	12	5	0	0	240	

Public safety and social service workers	186	76	51	23	3	1	240	

Single adults	96	40	117	49	25	11	240	

Adults with dependents	186	78	51	21	3	1	240	

Public officials	75	32	120	50	14	18	239	1

								

***Access to Hospital Treatment***								

The sickest patients	182	75	35	15	23	10	240	

Health care workers infected with pandemic flu while serving patients	203	85	33	14	4	2	240	

Elderly or chronically ill	76	32	103	42	61	26	240	

Public officials	57	24	121	51	61	25	239	1

Children	211	88	28	12	1	<0	240	

Single adults	84	35	122	51	34	15	240	

Adults with dependents	177	74	61	25	2	1	240	

A strong majority supported hospital treatment priorities being set (72% in favor versus 28% in opposition) while only a moderate majority favored each resident having an equal chance at antiviral access (59%). Subjects with < college/university education were significantly less likely (OR = 2.25, p < 0.05) to support the government setting priorities regarding distribution of antiviral medications. Females (OR = 2.3, p < 0.05) and those with < college/university education (OR = 2.98, p < 0.01) were more likely to agree that adults with chronic illnesses should have high priority to antiviral medications. In terms of limited treatment resources during a pandemic, older participants (OR = 2.56, p < 0.05) and females (OR = 2.51, p < 0.05) were more likely to believe the sickest patients should have high priority to hospital treatment. Females (OR = 2.19, p < 0.05) were less likely to believe that patients who were most likely to recover should have high priority access to hospital treatment. Non-married participants (OR = 2.84, p = 0.05) were more likely to believe that health care workers who become infected during a pandemic should have high priority to hospital treatment. Participants living in rural communities (OR = 3.13, p < 0.05) were less likely to believe that children should have high priority to hospital treatment access. Older participants and those with ≥ college/university education were less likely to believe that adults with dependents should have high priority to hospital treatment.

### Global Issues

Over 70% agreed that Canada should provide international assistance to poorer countries to prepare for a pandemic, even if resources for Canadians were reduced. While 92% of this group believed provision should be at least 7 to 10% of all resources generated, 43% believed the proportion should be greater than 10%. Unemployed individuals (OR = 3.14, p < 0.05) were more likely to disagree with providing international assistance and those unemployed (OR = 2.72, p < 0.05) and living in rural communities (OR = 3.00, p = 0.01) were significantly more likely to disagree with Canada donating more than 10% of total resources to poorer countries.

### Miscellaneous

Strong support was found for development of a plan enabling members of the public to play volunteer roles in maintaining order and offering services if properly trained (98%). Strong support was also found for having pandemic risks communicated by radio (90%), television (97%) and by the internet (81%), with somewhat less support for telephone communication (69%). The particular risks considered most relevant included knowing where the epidemic is most active (98%) and the current risks of death (69%) and infection (78%). Strong support was also found for how severe an offense the disobeying of quarantine orders should be considered. About half (50%) of respondents equated it with a manslaughter offense and over a quarter (27%) saw it as equal to physical assault. In terms of organization of citizens during a pandemic, subjects with < college/university education (OR = 1.83, p = 0.01) and with children (OR = 1.98, p < 0.05) were less likely to submit information into a national database enabling the government to request assistance (from them) during a pandemic. In terms of preferences for communicating health risk information, older participants (OR = 2.55, p < 0.05) were more likely to want health risk information communicated via internet. Females (OR = 1.87, p < 0.05) were more likely to want health risk information communicated via telephone. Younger participants were less likely to want to know risk of death (OR = 2.02, p < 0.05) and risk of infection (OR = 2.64, p = 0.01) (Figure 1).

## Discussion

This survey presents unique data on key issues in influenza pandemic response. Results indicate public consensus on several policy challenges, with strong support for: a) mortality reduction as the dominant goal of pandemic preparedness; b) restrictive measures being legitimate, providing restricted persons receive ample support; c) health care workers being obligated to provide care under pandemic conditions, if they receive adequate supports. Results further indicate respondents clearly recognize an obligation to assist other nations in their pandemic responses. One of the key majority views, the view that adequate protective antiviral dosages should be stocked for all Canadians (92%), is largely discrepant with current Canadian pandemic policy. In general we observed public values consistent with the ethical principal of solidarity. Canadians were willing to sacrifice liberties on behalf of the public good. However, in return they had expectations that public health and governmental officials would protect their interests.

The survey also indicates areas of sharp divergence and potential controversy, particularly in resource allocations and the response to health workers refusing to work during pandemics. These findings reinforce several in-depth normative differences in current ethical theory. For example, in priority setting there are conflicts between those who give priority to efficiency based approaches [5] and those who argue for protecting the most vulnerable, or not worsening their disadvantage [6,7]. Similarly, the empirical evidence that suggests a substantial proportion of health providers may not be prepared to work in a pandemic [8,9] varies markedly from the core values of health care professions and the prevailing contractual views of care provision [10-14]. Both key issues starkly illustrate the lack of consensus, empirically and normatively, informing several pandemic issues.

While surveys and other empirical studies have limitations in resolving normative issues, they provide important data, highlighting areas where policy setting can provoke strong public reactions. Surveys cannot replace argumentation and reflection, but they highlight areas where further public engagement is required and where decision-making authorities must be particularly explicit, transparent and accountable. This is especially the case for priority setting issues. In situations of scarcity and urgency, decisions will disadvantage some groups. This underlines the need for clear risk communication and strong leadership, and a process of post pandemic evaluation of all actions taken.

The results of this survey may be useful to public health authorities in planning pandemic responses. The observation of significant variances in responses per item suggests the instrument sensitivity to opinion differences. As such it can play a useful role in the dialogue on pandemic policy and other high risk situations where ethical issues are paramount.

This study is subject to several limitations, including modest sample size, given the goal of generalizing results to the Canadian population and a greater number of women surveyed than men. Nonetheless, the study had sufficient power to detect significant associations in regression analyses. While the randomization procedure was standard for RDD studies, there are noteworthy criticisms of RDD as access restrictions to land lines don't account for the increasing numbers who solely use cell phones and are not accessed via RDD. Furthermore, the surveys were conducted before the full impact of the current economic crisis and the emergence of the current H1N1 influenza strain. Our intent is to address such limitations in future studies.

The results reflect the Canadian public's trust in and high expectations of public health officials to make policy decisions that advance the public good even if this includes measures to restrict some individual freedoms (e.g. quarantine). The right of public officials to restrict individual freedoms is not absolute, but is accompanied by a corresponding obligation to meet conditions of reciprocity to minimize the burden of such restrictions on individual citizens.

## Conclusion

Surveys and other empirical studies provide important data on pandemic preparations, highlighting areas where policy setting can provoke strong reactions, where public engagement is additionally required and where authorities must be especially transparent and accountable. The Canadian public appears to trust officials with difficult decisions, providing reciprocity and respect for individual rights remain strongly emphasized.

## Competing interests

The authors declare that they have no competing interests.

## Authors' contributions

PR, KW, JLG, CST, JXN, ARJ, and REGU drafted survey items. CG, PR, and REGU conducted statistical analyses. CG, PR, KW, JLG, CST, JXN, ARJ, and REGU interpreted statistical analyses. PR, KW, JLG, CG, CST, JXN, ARJ, and REGU drafted the manuscript. PR, KW, CST, and REGU determined methodology. PR, KW, CST, and REGU supervised telephone survey administration. All authors read and approved the final manuscript.

## Pre-publication history

The pre-publication history for this paper can be accessed here:

http://www.biomedcentral.com/1471-2458/10/125/prepub
